# Trained hypertensive rats exhibit decreased transcellular vesicle trafficking, increased tight junctions’ density, restored blood-brain barrier permeability and normalized autonomic control of the circulation

**DOI:** 10.3389/fphys.2023.1069485

**Published:** 2023-02-23

**Authors:** Vanessa B. Candido, Sany M. Perego, Alexandre Ceroni, Martin Metzger, Alison Colquhoun, Lisete C. Michelini

**Affiliations:** ^1^ Department of Physiology and Biophysics, São Paulo, Brazil; ^2^ Department of Cell and Developmental Biology, Institute of Biomedical Sciences, University of Sao Paulo, São Paulo, São Paulo, Brazil

**Keywords:** aerobic exercise training, autonomic control, blood-brain barrier, transcytosis, paracellular transport, spontaneously hypertensive rats (SHR)

## Abstract

**Introduction:** Chronic hypertension is accompanied by either blood-brain barrier (BBB) leakage and autonomic dysfunction. There is no consensus on the mechanism determining increased BBB permeability within autonomic areas. While some reports suggested tight junction’s breakdown, others indicated the involvement of transcytosis rather than paracellular transport changes. Interestingly, exercise training was able to restore both BBB permeability and autonomic control of the circulation. We sought now to clarify the mechanism(s) governing hypertension- and exercise-induced BBB permeability.

**Methods:** Spontaneously hypertensive rats (SHR) and normotensive controls submitted to 4-week aerobic training (T) or sedentary protocol (S) were chronically cannulated for baseline hemodynamic and autonomic recordings and evaluation of BBB permeability. Brains were harvested for measurement of BBB function (FITC-10 kDa leakage), ultrastructural analysis of BBB constituents (transmission electron microscopy) and caveolin-1 expression (immunofluorescence).

**Results:** In SHR-S the increased pressure, augmented sympathetic vasomotor activity, higher sympathetic and lower parasympathetic modulation of the heart and the reduced baroreflex sensitivity were accompanied by robust FITC-10kDa leakage, large increase in transcytotic vesicles number/capillary, but no change in tight junctions’ density within the paraventricular nucleus of the hypothalamus, the nucleus of the solitary tract and the rostral ventrolateral medulla. SHR-T exhibited restored BBB permeability and normalized vesicles counting/capillary simultaneously with a normal autonomic modulation of heart and vessels, resting bradycardia and partial pressure reduction. Caveolin-1 expression ratified the counting of transcellular, not other cytoplasmatic vesicles. Additionally, T caused in both groups significant increases in tight junctions’ extension/capillary border.

**Discussion:** Data indicate that transcytosis, not the paracellular transport, is the primary mechanism underlying both hypertension- and exercise-induced BBB permeability changes within autonomic areas. The reduced BBB permeability contributes to normalize the autonomic control of the circulation, which suppresses pressure variability and reduces the occurrence of end-organ damage in the trained SHR. Data also disclose that hypertension does not change but exercise training strengthens the resistance of the paracellular pathway in both strains.

## 1 Introduction

Brain capillaries exhibit a unique restricted permeability compared to peripheral capillaries. The blood-brain barrier (BBB), a complex multicellular structure characteristic of brain capillaries, is composed of endothelial cells laying on the basement membrane which are enveloped by pericytes and astrocyte endfeet (Abbott et al., 2006; Daneman and Prat, 2015). Adjacent endothelial cells are mechanically linked together by tight junction protein complexes that limit the paracellular transport (Haseloff et al., 2015; Greene et al., 2019). In addition, brain endothelial cells exhibit unusually low levels of transcytosis and reduced trafficking between the circulating blood and the central nervous system (Ayloo and Gu, 2019).

Although the BBB is functionally competent in healthy individuals, several studies have shown BBB lesion in neurodegenerative diseases, stroke, trauma, dementia, and aging (Zlokovic, 2008; Faraco and Iadecola, 2013; Krueger et al., 2013; Rosenberg, 2014). BBB lesion was also detected in spontaneously hypertensive rats (SHR), allowing the entrance of plasma angiotensin II into the parenchyma of autonomic brain areas involved in cardiovascular control, which should be protected by the BBB (Biancardi et al., 2014).

In SHR we reported an intact BBB within autonomic areas of pre-hypertensive rats, but an important dysfunction accompanied by autonomic imbalance after the establishment of hypertension (Buttler et al., 2017). Interestingly, we demonstrated for the first time that exercise training was able to promptly reduce BBB leakage in SHR and normalize its function while correcting the autonomic dysfunction (Buttler et al., 2017). Although increased BBB leakage in chronic hypertension has been confirmed by several studies (Ueno et al., 2004; Pelisch et al., 2013; Biancardi et al., 2014; Mohammadi and Dehghani, 2014; Buttler et al., 2017), there is no consensus on the mechanism determining BBB permeability. Some studies pointed to BBB breakdown and paracellular transport changes (Pelisch et al., 2013; Biancardi et al., 2014; Mohammadi and Dehghani, 2014) while other indicated the transcellular, not the paracellular transport as the determinant of increased leakage (Ueno et al., 2004). Using caveolin-1 as a marker of transcytosis (caveolin-1 oligomerization is necessary for the expansion of caveolar invaginations and formation of transcellular vesicles, (Predescu et al., 1997; Zhao et al., 2014)), we suggested that both hypertension-induced BBB leakage and exercise-induced correction were caused by transcytosis across the BBB (Fragas et al., 2021). Although caveolin-1 changes in sedentary and trained SHR were positively correlated with BBB leakage, systolic arterial pressure variability and sympathetic vasomotor changes, they were only suggestive and did not prove the involvement of the transcellular transport.

In the present study we sought to confirm hypertension- and exercise-induced transcellular vesicle trafficking as the mechanism determining BBB permeability changes observed in sedentary and trained SHR. In addition, considering that nothing is known about the effects of exercise on paracellular transport across brain capillaries of hypertensive rats, we also investigated tight junctions (TJs) expression, as well as other structural changes in BBB within important autonomic areas including the paraventricular nucleus of the hypothalamus (PVN), the nucleus of the solitary tract (NTS) and the rostral ventrolateral medulla (RVLM). Transmission electron microscopy allowed us to evaluate hypertension- and exercise-induced ultrastructural BBB changes in brain capillaries of the SHR and age-matched normotensive controls, in order to correlate them with BBB permeability, hemodynamic changes and the autonomic control of the circulation. Special attention was given to the mechanisms controlling the transcellular and paracellular transport across the capillary endothelium.

## 2 Materials and Methods

### 2.1 Ethical approval, animals and experimental design

This study was carried out according the Ethical Principles in Animal Research of the Brazilian Council for Control of Animal Experimentation (CONCEA), in compliance with the ARRIVE guidelines. Surgical procedures and experimental protocols were reviewed and approved by the Institutional Animal Care and Use Committee of the Biomedical Sciences Institute, University of Sao Paulo (CEUA, protocol number 93/2017).

Chronically hypertensive male SHR (12-weeks old) and normotensive controls (Wistar) were housed in the Animal Facilities of Department of Physiology and Biophysics, University of São Paulo under controlled temperature/humidity, 12/12-h light/dark cycle with free access to standard chow and water. Rats body weight was measured throughout the experiment. During a 2-week adaptation period rats were pre-selected for their ability to walk/run on a treadmill (0.4–0.8 km/h, 0% grade, 10 min/day, Millenium, Inbramed, Porto Alegre, Brazil). Only active rats were included in this study. Rats were then submitted to progressive exercise tests (MET (Cavalleri et al., 2011; Fragas et al., 2021) to determine maximal individual aerobic capacity, to allocate rats with identical capacities to trained (T) and sedentary (S) groups and to set the intensity of aerobic training (T = 50–60% of maximal capacity, performed 1 h/day, 5 days/week for 4 week). Previous studies confirmed that 4 weeks of exercise training were enough to restore BBB permeability and induce cardiovascular adjustments in SHR (Masson et al., 2014; Buttler et al., 2017; Fragas et al., 2021). S groups were handled every day to approximate their conditions to those experienced by T groups. MET was performed again at the end of experimental protocols to evaluate the aerobic capacity of T and S groups (Fragas et al., 2021). SHR and Wistar rats were then pre-anesthetized with acepromazine (2.5 mg/kg *ip,* Syntec, Santana de Parnaiba, São Paulo, Brazil) followed by ketamine (80 mg/kg *ip,* Fort Dodge IA, United States of America) + xylazine (12 mg/kg *ip*, Alcon, Fort Worth TX, United States of America) for catheterization of the femoral artery. Rats were treated with penicillin (24,000IU/kg *im*, Pentabiotico Veterinario, Fontoura Wyeth, Brazil) and ketoprofen (2 mg/kg *sc*, Biofarm, Jaboticabal, Brazil) and return to their home cages for recovery, which extended for at least 24–30 h.

### 2.2 Functional/autonomic measurements

Baseline arterial pressure (AP) and heart rate (HR) were continuously acquired on a beat-to-beat basis (50–60 min, LabChart Pro, ADInstruments, sampling frequency of 2,000Hz) in conscious unrestrained rats resting in their home cages (Ichige et al., 2016). Time series of systolic AP (SAP) and pulse interval (PI) were used to evaluate pressure and HR variabilities at the frequency domain (Buttler et al., 2017; Fragas et al., 2021). Power spectral density for the low frequency (LF, 0.20–0.75 Hz, indicative of sympathetic vasomotor activity and sympathetic + parasympathetic activity to the heart), the high frequency (HF, 0.75–3.00 Hz, indicative of cardiac vagal modulation) and very low frequency (VLF, <0.20 Hz, suggestive of hormonal modulation) were evaluated (Masson et al., 2014). LF/HF ratio to the heart and spontaneous baroreflex sensitivity were also calculated.

### 2.3 Analysis of BBB integrity/lesion

After hemodynamic recordings a subgroup of SHR-S, SHR-T, Wistar-S, and Wistar-T was again anesthetized for catheterization of right carotid artery. A mixture of dyes [rhodamine isothiocyanate dextran, 70 kDa (RHO) and fluorescein isothiocyanate dextran 10 kDa (FITC], Sigma-Aldrich) was slowly administered and allowed to recirculate as previously described (Biancardi et al., 2014). Rats received then and overdose of anesthesia (300 mg/kg ketamine +60 mg/kg xylazine *ip*) for brain harvesting immediately after the respiratory arrest. Brains were post-fixed (4% phosphate-buffered paraformaldehyde, 48 h), cryoprotected (30% sucrose in PBS for 72 h) and stored until processing (Buttler et al., 2017; Rocha-Santos et al., 2020).

Sequential coronal PVN, NTS and RVLM slices (30 μm, Leica CM1850 cryostat, Germany) were collected and mounted in gelatinized slides as previously described (Buttler et al., 2017). The BBB permeability was analyzed by the quantitative assessment of intravascular and extravascular dyes according the technique developed by Biancardi et al. (2014). With an intact BBB both dyes are colocalized within brain capillaries; in the presence of compromised barrier integrity the large-size dye are still contained by the capillaries whereas the small-size dye partially leaks into the brain parenchyma (Biancardi et al., 2014). Tissues were examined by a blind observer on a fluorescent microscope (Leica BMLB, Nussloch, Germany) attached to an Exiblue camera (Imaging, Canada). Selected images were acquired by Image-Pro Plus software (Media Cybernetics, United States) and quantified by the ImageJ software (NIH, United States).

### 2.4 Ultrastructural analysis of the BBB constituents

Another subgroup of SHR-S, SHR-T, Wistar-S, and Wistar-T received, after the functional measurements, an overdose of ketamine + xylazine. Immediately after the respiratory arrest, the thorax was opened and the left ventricle cannulated for sterile saline perfusion (∼30 mL/min, Daigger pump, Vernon Hills IL United States) followed by modified Karnovsky solution (2.5% glutaraldehyde +2% paraformaldehyde in 0.1 M PBS, pH 7.3). Brain was removed and placed on a coronal brain matrix (72–5029, Harvard Apparatus) to obtain hypothalamic and brainstem slices. PVN, NTS, and RVLM nuclei were microdissected with the aid of a magnifying lens, using as anatomic markers the third ventricle and optic chiasma, the central canal and 4th ventricle, and, the nucleus ambiguous, raphe obscurus and inferior olive, respectively. The nuclei were immersed in a 2.5% glutaraldehyde solution for 2 h, washed in PBS and post-fixed in a 2% osmium tetroxide solution for 2 h at 4°C. Tissues were then stained overnight with uranyl acetate, dehydrated in 60% up to 100% ethanol series and immersed in pure resin. Semi-thin slices (400 nm, ultra-microtome Leica EMUC6) were obtained, placed in glass slides and stained with Toluidine Blue in order to select adequate areas for further processing. Ultra-thin slices (60 nm) were obtained with diamond knife, contrasted with 4% uranyl acetate and 0.4% lead acetate and disposed in 200 copper mesh screens.

Transverse sections of PVN, NTS, and RVLM capillaries of the 4 experimental groups were acquired in a transmission electron microscope (FEI Tecnai G20, 200 KV) and analyzed by a blind observer using the ImageJ software. The following parameters were analyzed in 9–11 capillaries/area/rat, 3 rats/experimental group: luminal and abluminal perimeter, lumen diameter, area of the endothelial cell, thickness of the basement membrane, pericytes’ coverage of capillaries, extension of capillary border between adjacent endothelial cells, the occurrence/extension of tight junctions, and, the counting of transcellular vesicles/capillary. To avoid the inclusion of non-transcytotic vesicles such as lysosomes, endosomes, peroxisomes, only the vesicles being formed at the luminal, and abluminal membranes were counted. Vesicle counting was expressed as number/capillary. Using the zoom to expand acquired images, the whole extension of capillaries was analyzed.

### 2.5 Immunofluorescence assays

Some rats of each experimental group were used to confirm our counting of transcytotic vesicles by means of caveolin-1 expression. Caveolin-1, the main component of transcellular vesicle membranes (Predescu et al., 1997; Zhao et al., 2014) was used as a marker of transcytosis. After the functional measurements rats received an overdose of ketamine + xylazine. Immediately after the respiratory arrest brains were harvested, post-fixed, cryoprotected and stored as previously described (Fragas et al., 2021).

For caveolin-1 immunofluorescence assay coronal PVN sections (30 μm) were collected (Fragas et al., 2021). Briefly, free-floating sections were incubated with a mixture of primary antibodies (rabbit anti-caveolin-1, Cell Signaling 1:100 dilution + mouse-anti-endothelial cell antibody RECA-1, Abcam, 1:800 dilution for 48 h at 4°C) followed by incubation with secondary antibodies (anti-rabbit Alexa Fluor 488 + anti-mouse Alexa Fluor 549, Jackson ImmunoResearch, 1:500 dilution each, at room temperature for 1 h). Sections, mounted in gelatinized slides, were examined by a blind observer in a fluorescence microscope (Axioimager AI, Zeiss, Munchen, Germany attached to a Zeiss Axiocam 512 camera). Caveolin-1 immunofluorescence was normalized by RECA-1 immunoreactivity, both signals being acquired in the same ROI. Images were acquired with identical acquisition settings and analyzed as previously described (Rocha-Santos et al., 2020). Values of several PVN slices (expressed as integrated density) were averaged to yield a mean value/rat.

### 2.6 Evaluation of cardiac hypertrophy

Some rats of each group were, at the end of functional experiments, euthanized with an overdose of ketamine + xylazine. Immediately after the respiratory arrest the thorax was opened and the heart removed to isolate the left ventricle which was weighed in a semi-analytical scale (Micronal B400, SP, Brazil). The ratio of left ventricle/body weight was used as an index of cardiac hypertrophy.

### 2.7 Statistical analysis

Data are presented as means ± SEM and submitted to Shapiro-Wilk homogeneity variance test. Treadmill performance and body weight in S and T SHR and Wistar rats were analyzed by three-way ANOVA with repeated measurements (time). Differences in hemodynamic and autonomic parameters, BBB permeability, ultrastructural changes in BBB constituents, and caveolin-1 expression between groups and conditions were analyzed by two-way factorial ANOVA. Tukey was the *post hoc* test. Correlations analyses used the Pearson statistics. All statistical analyses were performed by the GraphPad Prism 8 software. Differences were considered significant at *p* < 0.05.

## 3 Results

### 3.1 Treadmill performance and functional data

SHR had smaller body weight and better treadmill performance than respective age-matched controls during the experimental protocols (Table 1). SHR-S, SHR-T, Wistar-S and Wistar-T exhibited similar body weight gain during the 4-week protocols. Both trained groups showed a significant and similar performance gain after 4 weeks of daily exercise while sedentary groups exhibited a small decrease (SHR-S) or no change (Wistar-S) (Table 1).

**TABLE 1:** Treadmill performance and body weight during experimental protocols, resting hemodynamic parameters, autonomic modulation and cardiac hypertrophy at the end of protocols in sedentary (S) and trained (T) Wistar and spontaneously hypertensive rats (SHR).

	Wistar-S	Wistar-T	SHR-S	SHR-T
***Treadmill performance***	***n = 20***	***n = 20***	***n = 20***	***n = 20***
***week 0*** (*km/h*)	1.01 ± 0.05	1.01 ± 0.04	1.31 ± 0.04*	1.31 ± 0.05*
***week 4*** (*km/h*)	0.81 ± 0.03	1.40 ± 0.04^**#**^ **†**	0.92 ± 0.07^**#**^	1.83 ± 0.09^#^*†
***gain*** (*% change*)	−20%	+39%	−30%	+40%

***Body Weight***	***n = 20***	***n = 20***	***n = 20***	***n = 20***
***week 0*** *(g)*	376 ± 6	377 ± 5	294 ± 6*	289 ± 6*
***week 4*** *(g)*	433 ± 6^**#**^	409 ± 6^**#**^	328 ± 7^#^*	318 ± 6^#^*
***gain*** (*% change*)	+15%	+9%	+12%	+10%

***Hemodynamic parameters***	***n = 18***	***n = 17***	***n = 17***	***n = 18***
***MAP*** (*mmHg*)	109 ± 2	109 ± 4	170 ± 4*	155 ± 4*†
***HR*** (*b/min*)	352 ± 5	327 ± 5**†**	367 ± 7	337 ± 5**†**

***Power Spectral Analysis***	***n = 18***	***n = 17***	***n = 17***	***n = 18***
***SAP variability*** (*mmHg* ^*2*^)	21.65 ± 3.10	17.14 ± 1.38	42.18 ± 3.80*	31.41 ± 3.03*
***LF-SAP*** (*mmHg* ^*2*^)	5.77 ± 0.57	5.46 ± 0.56	9.17 ± 1.49*	6.22 ± 0.59
***VLF-SAP*** (*mmHg* ^*2*^)	7.70 ± 1.47	6.01 ± 0.97	18.01 ± 2.23*	12.21 ± 1.16*†
***PI variability*** (*ms* ^*2*^)	33.12 ± 4.41	37.71 ± 4.36	18.95 ± 2.63*	34.12 ± 2.78†
***LF-PI*** (*ms* ^*2*^)	5.77 ± 0.57	5.46 ± 0.56	10.46 ± 1.93*	6.22 ± 0.59†
***HF-PI*** (*ms* ^*2*^)	7.96 ± 0.83	8.14 ± 0.83	5.06 ± 0.52*	9.89 ± 0.79†
***LF/HF ratio***	0.40 ± 0.05	0.41 ± 0.05	0.59 ± 0.02*	0.28 ± 0.02†
***αLF*** (*ms/mmHg*)	0.84 ± 0.06	1.04 ± 0.07	0.42 ± 0.06*	0.68 ± 0.06*†
***αHF*** (*ms/mmHg*)	1.93 ± 0.17	2.71 ± 0.20†	1.60 ± 0.10	2.36 ± 0.10†
***LVW/BW ratio*** (*mg/g*)	1.84 ± 0.04	1.94 ± 0.04	2.74 ± 0.13*	2.89 ± 0.16*

Values are means ± SEM. MAP, mean arterial pressure; HR, heart rate; SAP, systolic arterial pressure; LF, low frequency component; HF, high frequency component; VLF, very-low frequency component; PI, pulse interval; αLF and αHF, spontaneous baroreflex sensitivity calculated with LF-PI and HF-PI components respectively, in relation to pressure changes; LVW, left ventricle weight; BW, body weight. Treadmill performance and body weight comparisons made by Three-way ANOVA for repeated measurements (time). Treadmill Performance: group F (1,76) = 55.59, p < 0.001, condition F (1,76) = 96.25, p < 0.001, time F (1,76) = 4.57, p = 0.036, group x time F (1,76) = 0.15, p = 0.699, condition x time F (1,76) = 94.34, p < 0.001, group x condition F (1,76) = 4.66, p = 0.034, group x condition x time F (1,76) = 4.57, p = 0.036; Body Weight: group F (1,76) = 250.2, p < 0.001, condition F (1,76) = 2.72, p = 0.103, time F (1,76) = 489.9, p < 0.001, group x time F (1,76) = 14.75, p < 0.001, condition x time F (1,76) = 19.16, p < 0.001, group x condition F (1,76) = 0.13, p = 0.721, group x condition x time F (1,76) = 8.56, p = 0.005. Other comparisons made by Factorial Two-way ANOVA. MAP: group F (1,66) = 228.8, p < 0.001, condition F (1,66) = 4.38, p = 0.040, group x condition F (1,66) = 4.38, p = 0.040; HR: group F (1,66) = 5.09 p = 0.027, condition F (1,66) = 24.63, p < 0.001, group x condition F (1,66) = 0.20, p = 0.653; SAP variability: group F (1,66) = 34.27, p < 0.001, condition F (1,66) = 6.61, p = 0.012, group x condition F (1,66) = 1.11, p = 0.296; LF-SAP: group F (1,66) = 5.58, p = 0.021, condition F (1,66) = 3.43, p = 0.068, group x condition F (1,66) = 2.25, p = 0.138; VLF-SAP: group F (1,66) = 29.33, p < 0.001, condition F (1,66) = 6.01, p = 0.017, group x condition F (1,66) = 1.82, p = 0.182; PI variability: group F (1,66) = 5.92, p = 0.018, condition F (1,66) = 7.33, p = 0.009, group x condition F (1,66) = 2.10, p = 0.152; LF-PI: group F (1,66) = 6.57, p = 0.013, condition F (1,66) = 4.58, p = 0.036, group x condition F (1,66) = 3.42, p = 0.069; HF-PI: group F (1,66) = 0.58, p = 0.450, condition F (1,66) = 10.93, p = 0.002, group x condition F (1,66) = 9.42, p = 0.003; LF/HF ratio: group F (1,66) = 0.62, p = 0.434, condition F (1,66) = 15.49, p < 0.001, group x condition F (1,66) = 17.63, p < 0.001; αLF: group F (1,66) = 38.88, p < 0.001, condition F (1,66) = 13.52, p = 0.001, group x condition F (1,66) = 0.23, p = 0.633; αHF: group F (1,66) = 5.23, p = 0.025, condition F (1,66) = 26.83, p < 0.001, group x condition F (1,66) = 0.01, p = 0.945; LVW/BW: group F (1,50) = 200.4, p < 0.001, condition F (1,50) = 4.45, p = 0.040, group x condition F (1,50) = 0.03, p = 0.881. Significances (p < 0.05) are # vs. week 0, * vs. respective WKY, † vs. respective S rats*.*

At the end of protocols MAP (+56%), SAP variability (+95%), vasomotor and cardiac sympathetic activities (+59% and +81% for LF-SAP and LF-PI, respectively) and hormonal modulation (VLF-SAP, +134%) were markedly increased in SHR-S vs*.* Wistar-S (Table 1). In contrast, there were significant decreases in PI variability (−43%) and parasympathetic modulation of the heart (HF-PI, −36%) in such a way that cardiac sympathovagal balance was augmented and spontaneous baroreflex sensitivity reduced in SHR-S vs. Wistar-S (Table 1). These changes were also accompanied by significant cardiac hypertrophy (2.74 ± 0.13 and 1.84 ± 0.04 mg/g for SHR-S and Wistar-S, respectively)*.* On the other hand, SHR-T exhibited reduced MAP and resting HR (−9% and −8%) which were accompanied by large reductions in SAP variability and hormonal modulation and an almost normalized autonomic control of the circulation (data on Table 1). Spontaneous baroreflex sensitivity was increased in SHR-T vs. SHR-S, even in the presence of maintained cardiac hypertrophy (SHR-T = 2.89 ± 0.16 mg/g). Wistar rats responded to training with resting bradycardia and a mild increase in spontaneous baroreflex sensitivity (Table 1).

### 3.2 Hypertension- and exercise-induced changes in BBB permeability

**FIGURE 1:**
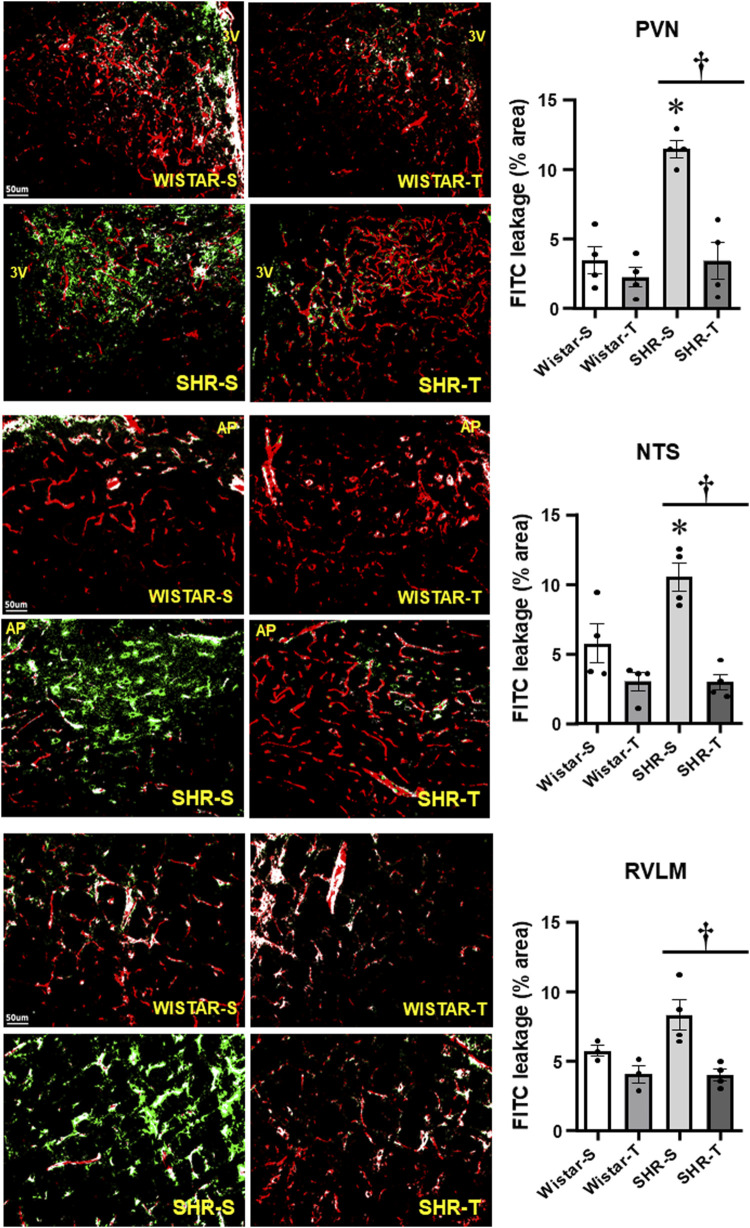
Comparison of BBB permeability within the PVN, NTS, and RVLM of sedentary (S) and trained (T) SHR and Wistar rats. Left photomicrographs show the capillary network (Rhodamine-70kDa, red), the FITC-10kDa leakage (green) into the brain parenchyma and the colocalization of both inside capillaries (white) within the three autonomic areas. Scale bars = 50 μm. Right bar graphs depict the effects of hypertension and exercise training on the BBB leakage into the PVN, NTS and RVLM. Values are means of 5-7 slices/area/rat, 3-4 rats/group. Comparisons made by 2-way factorial ANOVA. PVN: group F (1,12) = 23.57, p < 0.001, condition F (1,12) = 23.98, p < 0.001, group x condition F (1,12) = 13.06, p = 0.004; NTS: group F (1,12) = 5.96, p = 0.031, condition F (1,12) = 28.53, p < 0.001, group x condition F (1,12) = 6.27, p = 0.028; RVLM: group F (1,10) = 3.40, p = 0.090, condition F (1,10) = 19.79, p < 0.001, group x condition F (1,10) = 3.85, p = 0.073. Significances (p < 0.05) are * vs. respective Wistar group; † vs. respective S control.

BBB permeability was evaluated in anesthetized rats by the amount of FITC leakage into the brain parenchyma after hemodynamic and autonomic recordings. Hypertension was accompanied by marked BBB dysfunction, characterized by a huge leakage into the three autonomic areas (11.44 ± 0.61, 10.52% ± 1.02% and 8.31% ± 1.09% area for the PVN, NTS, and RVLM, respectively, corresponding to increases of 3.3-, 1.8- and 1.5-fold when compared with respective Wistar-S controls, Figure 1). Notice that exercise training not only reduced, but completely normalized BBB leakage within the three autonomic areas. Mild non-significant FITC leakage reductions were observed in Wistar-T vs. Wistar-S rats (Figure 1).

### 3.3 Hypertension- and exercise-induced changes in BBB ultrastructure

To uncover the mechanisms conditioning hypertension- and training-induced BBB permeability changes we analyzed, in the experimental groups, the ultrastructure of brain capillaries within autonomic areas (Table 2). Hypertension was accompanied by significant increases in capillaries’ lumen diameter within the three areas analyzed (+31%, +15% and +17%, SHR-S vs. Wistar-S, for PVN, NTS, and RVLM, respectively, *p* < 0.05), a change that was corrected by exercise training in the NTS and RVLM, but not in the PVN whose capillaries’ lumen were still distended in the SHR-T (Table 2). Except for the RVLM that exhibited a reduction (−19% in SHR-S vs. Wistar-S, *p* < 0.05), the basement membrane thickness was not changed by hypertension. Exercise training increased capillaries’ membrane thickness only in the NTS of Wistar rats, with no changes within autonomic areas of the other groups (Table 2).

**TABLE 2:** Quantitative data of PVN, NTS, and RVLM capillaries’ ultrastructure in the 4 experimental groups.

	Wistar-S	Wistar –T	SHR-S	SHR-T
PVN				
Luminal perimeter (µm)	17.44 ± 0.55	18.98 ± 0.87	22.81 ± 0.75*	24.46 ± 1.20*
Abluminal perimeter (µm)	19.28 ± 0.58	21.49 ± 0.98†	25.97 ± 0.87*	27.28 ± 1.23
Lumen diameter (µm)	5.55 ± 0.18	6.04 ± 0.28	7.26 ± 0.24*	7.79 ± 0.38*
Endothelial cell area (µm^2^)	5.63 ± 0.48	7.22 ± 0.61	12.04 ± 1.26*	9.55 ± 0.80
Basement membrane thickness (µm)	0.072 ± 0.002	0.080 ± 0.003	0.075 ± 0.004	0.064 ± 0.003*
Transcytotic vesicles/capillary (n)	4.69 ± 0.43	3.00 ± 0.46	8.03 ± 1.00*	3.61 ± 0.60†
Capillary border extension (µm)	1.39 ± 0.17	0.81 ± 0.09†	1.25 ± 0.13	1.09 ± 0.12
Tight junction extension (µm)	0.54 ± 0.08	0.56 ± 0.06	0.60 ± 0.07	0.74 ± 0.10
Tight junction/capillary border (%)	40 ± 4	72 ± 4†	47 ± 4	67 ± 6†
Pericytes’ coverage (%)	31 ± 2	43 ± 3†	29 ± 3	29 ± 3*
**NTS**				
Luminal perimeter (µm)	17.17 ± 0.49	18.51 ± 0.67	19.73 ± 0.85*	17.27 ± 0.53
Abluminal perimeter (µm)	19.49 ± 0.52	21.43 ± 0.75	22.77 ± 0.99*	19.42 ± 0.61†
Lumen diameter (µm)	5.46 ± 0.16	5.89 ± 0.22	6.28 ± 0.27*	5.50 ± 0.17
Endothelial cell area (µm^2^)	7.02 ± 0.65	9.15 ± 0.86	10.18 ± 1.09*	6.24 ± 0.71†
Basement membrane thickness (µm)	0.068 ± 0.003	0.090 ± 0.004†	0.061 ± 0.002	0.073 ± 0.006*
Transcytotic vesicles/capillary (n)	4.93 ± 0.63	3.96 ± 0.57	7.29 ± 0.98	4.13 ± 0.60†
Capillary border extension (µm)	0.87 ± 0.10	1.13 ± 0.12	1.32 ± 0.16	1.06 ± 0.12
Tight junction extension (µm)	0.43 ± 0.06	0.71 ± 0.06†	0.53 ± 0.10	0.68 ± 0.08
Tight junction/capillary border (%)	46 ± 5	66 ± 4†	39 ± 4	65 ± 4†
Pericytes’ coverage (%)	24 ± 2	40 ± 4†	26 ± 3	25 ± 3*
**RVLM**				
Luminal perimeter (µm)	15.69 ± 0.43	16.04 ± 0.44	18.29 ± 0.66*	16.95 ± 0.73
Abluminal perimeter (µm)	17.75 ± 0.49	19.02 ± 0.50	20.27 ± 0.71*	18.64 ± 0.78
Lumen diameter (µm)	4.99 ± 0.14	5.11 ± 0.14	5.82 ± 0.21*	5.40 ± 0.23
Endothelial cell area (µm^2^)	5.55 ± 0.60	8.16 ± 0.84†	6.28 ± 0.59	5.35 ± 0.46*
Basement membrane thickness (µm)	0.072 ± 0.002	0.078 ± 0.002	0.058 ± 0.002*	0.063 ± 0.003*
Transcytotic vesicles/capillary (n)	4.53 ± 0.59	4.13 ± 0.50	7.44 ± 0.85*	3.96 ± 0.77†
Capillary border extension (µm)	1.28 ± 0.16	1.11 ± 0.11	1.22 ± 0.22	1.30 ± 0.26
Tight junction extension (µm)	0.71 ± 0.11	0.77 ± 0.09	0.78 ± 0.17	0.90 ± 0.16
Tight junction/capillary border (%)	55 ± 4	71 ± 5	60 ± 5	71 ± 5
Pericytes’ coverage (%)	33 ± 2	26 ± 2	28 ± 3	34 ± 3

Values are means ± SEM. n = 25–30 capillaries/group, 3 rats/group. Comparisons made by Factorial Two-way ANOVA. PVN Luminal perimeter: group F (1,108) = 41.08, p < 0.001, condition F (1,108) = 3.55, p = 0.062, group x condition F (1,108) = 0.01, p = 0.948; Abluminal perimeter: group F (1,108) = 26.61, p < 0.001, condition F (1,108) = 12.59, p = 0.001, group x condition F (1,108) = 4.50, p = 0.036; Lumen diameter: group F (1,108) = 40.78, p < 0.001, condition F (1,108) = 3.54, p = 0.062, group x condition F (1,108) = 0.01, p = 0.948; Endothelial cell area: group F (1,108) = 26.67, p < 0.001, condition F (1,108) = 0.28, p = 0.596, group x condition F (1,108) = 5.81, p = 0.018; Basement membrane thickness: group F (1,108) = 4.44, p = 0.038, condition F (1,108) = 0.24, p = 0.628, group x condition F (1,108) = 9.47, p = 0.003; Transcytotic vesicles/capillary: group F(1,108) = 8.70, p = 0.004, condition F(1,108) = 20.83, p < 0.001, group x condition F(1,108) = 4.16, p = 0.044; Capillary border extension: group F (1,88) = 0.25, p = 0.618, condition F (1,88) = 7.02, p = 0.010, group x condition F (1,88) = 2.26, p = 0.136; Tight junction extension: group F (1,88) = 2.28, p = 0.134, condition F (1,88) = 1.02, p = 0.317, group x condition F (1,88) = 0.57, p = 0.452; Tight junction/capillary border: group F (1,88) = 0.02, p = 0.877, condition F (1,88) = 31.96, p < 0.001, group x condition F (1,88) = 1.61, p = 0.208; Pericytes coverage: group F (1,102) = 9.81, p = 0.002, condition F (1,102) = 4.61, p = 0.034, group x condition F (1,102) = 4.30, p = 0.041; NTS: Luminal perimeter: group F (1,116) = 1.03, p = 0.312, condition F (1,116) = 0.74, p = 0.391, group x condition F (1,116) = 8.53, p = 0.004; Abluminal perimeter: group F (1,116) = 0.74, p = 0.392, condition F (1,116) = 0.91, p = 0.342, group x condition F (1,116) = 12.81, p < 0.001; Lumen diameter: group F (1,116) = 1.08, p = 0.301, condition F (1,116) = 0.71, p = 0.400, group x condition F (1,116) = 8.54, p = 0.004; Endothelial cell area: group F (1,116) = 0.02, p = 0.883, condition F (1,116) = 1.15, p = 0.286, group x condition F (1,116) = 12.91, p < 0.001; Basement membrane thickness: group F (1,116) = 8.86, p = 0.004, condition F (1,116) = 17.78, p < 0.001, group x condition F (1,116) = 1.54, p = 0.217; Transcytotic vesicles/capillary: group F (1,116) = 3.13, p = 0.079, condition F (1,116) = 8.35, p = 0.005, group x condition F (1,116) = 2.35, p = 0.128; Capillary border extension: group F (1,106) = 2.27, p = 0.135, condition F (1,106) = 0.00, p > 0.999, group x condition F (1,106) = 4.25, p = 0.042; Tight junction extension: group F (1,106) = 0.42, p = 0.839, condition F (1,106) = 10.20, p = 0.002, group x condition F (1,106) = 0.37, p = 0.542; Tight junction/capillary border: group F (1,106) = 1.42, p = 0.237, condition F (1,106) = 31.43, p < 0.001, group x condition F (1,106) = 0.71, p = 0.401; Pericytes coverage: group F (1,112) = 4.40, p = 0.038, condition F (1,112) = 5.78, p = 0.018, group x condition F (1,112) = 6.73, p = 0.011; RVLM Luminal perimeter: group F (1,110) = 9.32, p = 0.003, condition F (1,110) = 0.74, p = 0.391, group x condition F (1,110) = 2.16, p = 0.145; Abluminal perimeter: group F (1,110) = 2.90, p = 0.091, condition F (1,110) = 0.08, p = 0.775, group x condition F (1,110) = 5.33, p = 0.023; Lumen diameter: group F (1,110) = 9.37, p = 0.003, condition F (1,110) = 0.67, p = 0.414, group x condition F (1,110) = 2.18, p = 0.143; Endothelial cell area: group F (1,110) = 2.65, p = 0.106, condition F (1,110) = 1.73, p = 0.191, group x condition F (1,110) = 7.67, p = 0.007; Basement membrane thickness: group F (1,110) = 40.95, p < 0.001, condition F (1,110) = 5.89, p = 0.017, group x condition F (1,110) = 0.05, p = 0.826; Transcytotic vesicles/capillary: group F (1,110) = 3.93, p = 0.049, condition F (1,110) = 7.88, p = 0.006, group x condition F (1,110) = 4.97, p = 0.028; Capillary border extension: group F (1,89) = 0.11, p = 0.742, condition F (1,89) = 0.05, p = 0.820, group x condition F (1,89) = 0.40, p = 0.527; Tight junction extension: group F (1,89) = 0.48, p = 0.488, condition F (1,89) = 0.39, p = 0.5533, group x condition F (1,89) = 0.04, p = 0.835; Tight junction/capillary border: group F (1,89) = 0.31, p = 0.578, condition F (1,89) = 7.90, p = 0.006, group x condition F (1,89) = 0.19, p = 0.662; Pericytes coverage: group F (1,110) = 0.27, p = 0.607, condition F (1,110) = 0.01, p = 0.918, group x condition F (1,110) = 7.05, p = 0.009. Significances (p < 0,05) * vs. respective Wistar, † vs. respective S.

**FIGURE 2:**
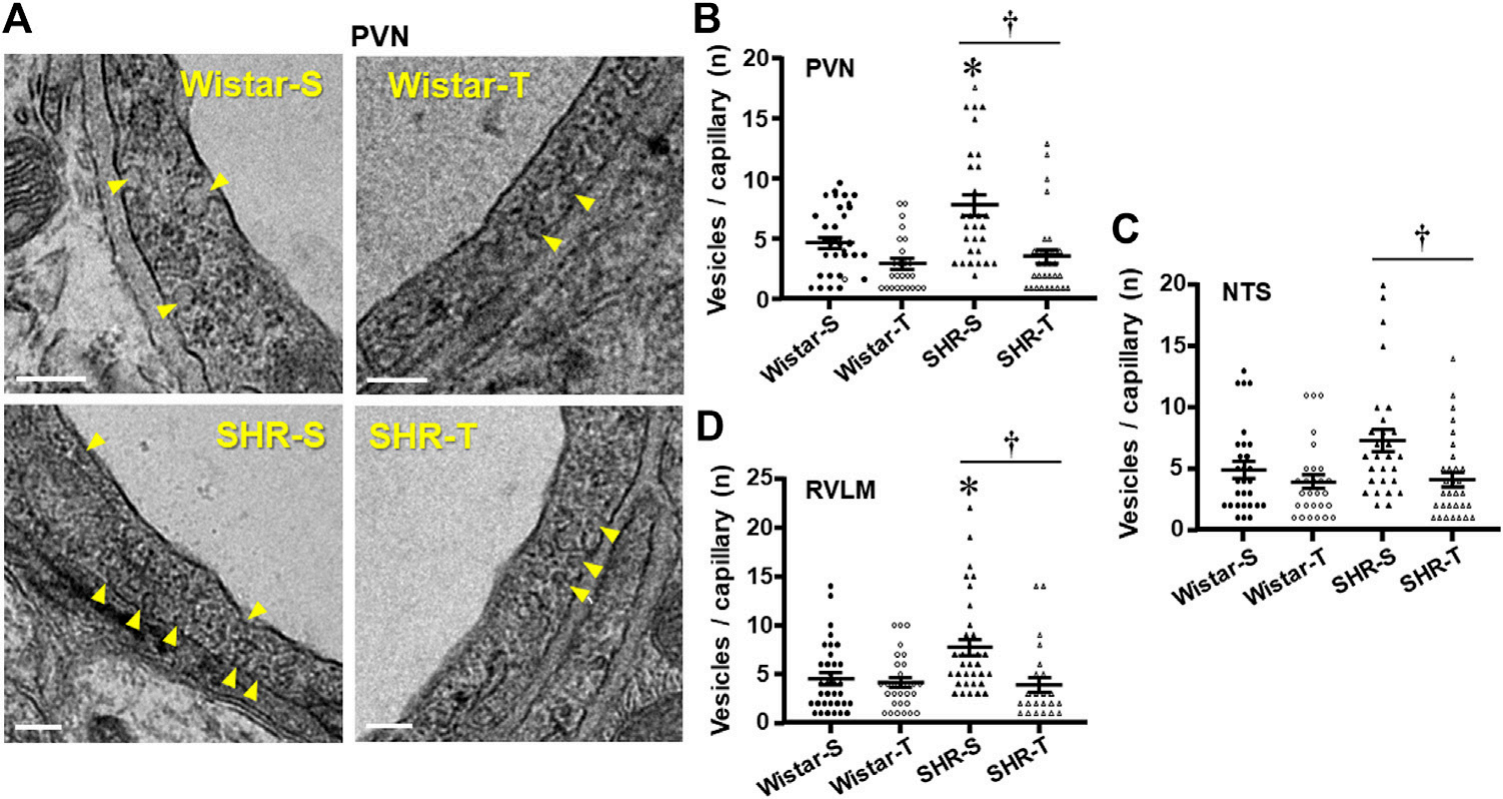
Effects of hypertension and exercise training on transcellular vesicles number/capillary. **(A)**. Electron micrographs depicting the transcellular vesicles (yellow arrows) being formed in the luminal and abluminal borders of the endothelial cell within PVN capillaries of sedentary (S) and trained (T) SHR and Wistar rats. Scale bars = 200 nm Right panels show the quantification of vesicle number in the 4 experimental groups at the end of protocols within the PVN **(B)**, NTS **(C)**, and RVLM **(D)** capillaries. *n* = 9–11 capillaries/rat, 3 rats/group. Comparisons made by 2-way factorial ANOVA. Significances (*p* < 0.05) are * vs. respective Wistar group; † vs. respective S control.

Luminal and abluminal vesicle number within the endothelium of PVN capillaries was increased by hypertension, but reduced after exercise training in both groups, with a large effect in hypertensive rats (Figure 2A). Quantitative data confirmed this observation showing a 71% augmentation in vesicles/capillary in SHR-S vs. Wistar-S (Figure 2B). Interestingly, the vesicle number was greatly reduced by training in SHR-T, showing a value similar to those exhibited by normotensive rats. The smaller reduction in vesicle number observed in Wistar-T vs. Wistar-S did not attain significance (Figure 2B). Similar hypertension- and training-induced effects on vesicle number were observed in both NTS and RVLM capillaries (Figures 2C, D; Supplementary Figure S1). It was also observed that the increased transcytosis within autonomic brain areas of the SHR-S was accompanied by a larger endothelial cell area, an effect that was reversed by exercise training (Table 2).

**FIGURE 3:**
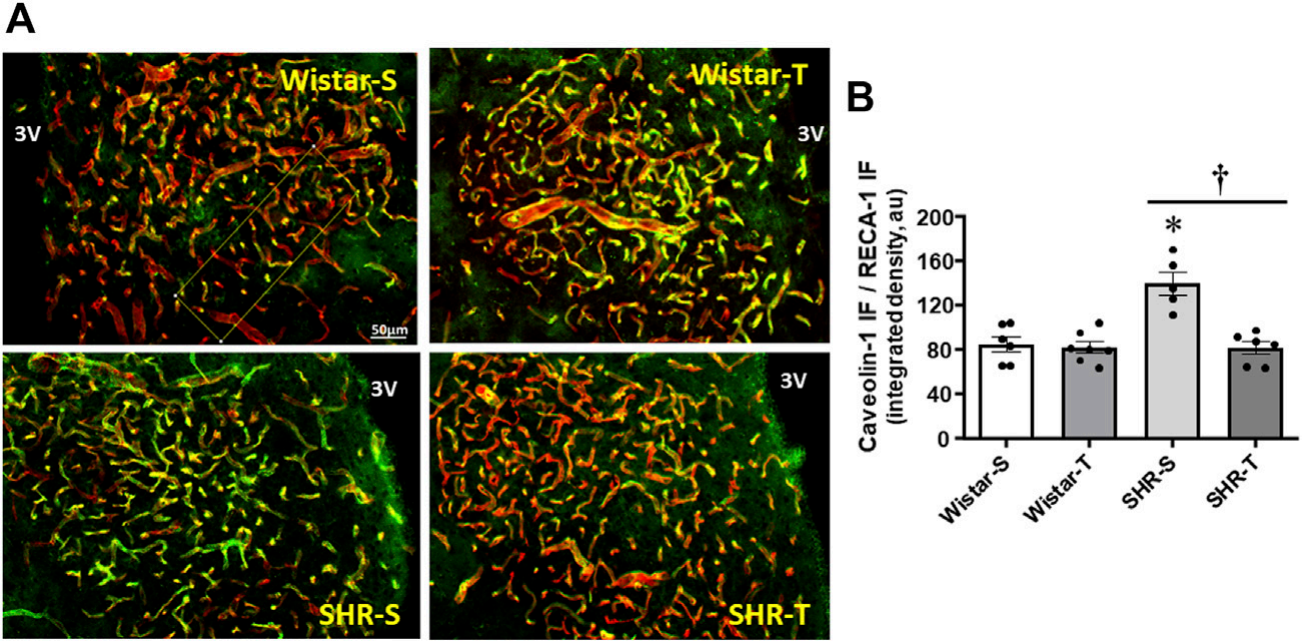
Effects of hypertension and exercise training on caveolin-1 content within the PVN. **(A).** Photomicrographs show caveolin-1 expression (green) within PVN capillaries (RECA-1, red) and the colocalization of both (yellow) in sedentary (S) and trained (T) SHR and Wistar rats. Scale bar = 50 μm, 3V, third ventricle. **(B).** Comparison of PVN caveolin-1 immunofluorescence in the 4 experimental groups at the end of protocols. *n* = 5–7 slices/rat, 6 rats/group. Comparisons made by 2-way factorial ANOVA: group F (1,20) = 13.36, p = 0.002, condition (F1,20) = 19.03, p < 0.001, group x condition (F1,20) = 13.74, p = 0.001. Significances (p < 0.05) are * vs. respective Wistar group; † vs. respective S control.

To corroborate the electron microscopy measurements of transcellular, not other cytoplasmatic vesicles, we analyzed the effects of hypertension and exercise on caveolin-1 expression, the main component of caveolae-mediated transcytosis. Within PVN capillaries (marked by RECA-1 antibody) caveolin-1 density was 61% higher in SHR-S (vs. Wistar-S), but largely reduced in SHR-T, attaining a value similar to those exhibited by normotensive groups (Figures 3A, B). Exercise training caused only a slight not significant reduction of caveolin-1 density in Wistar-T vs. Wistar-S.

**FIGURE 4:**
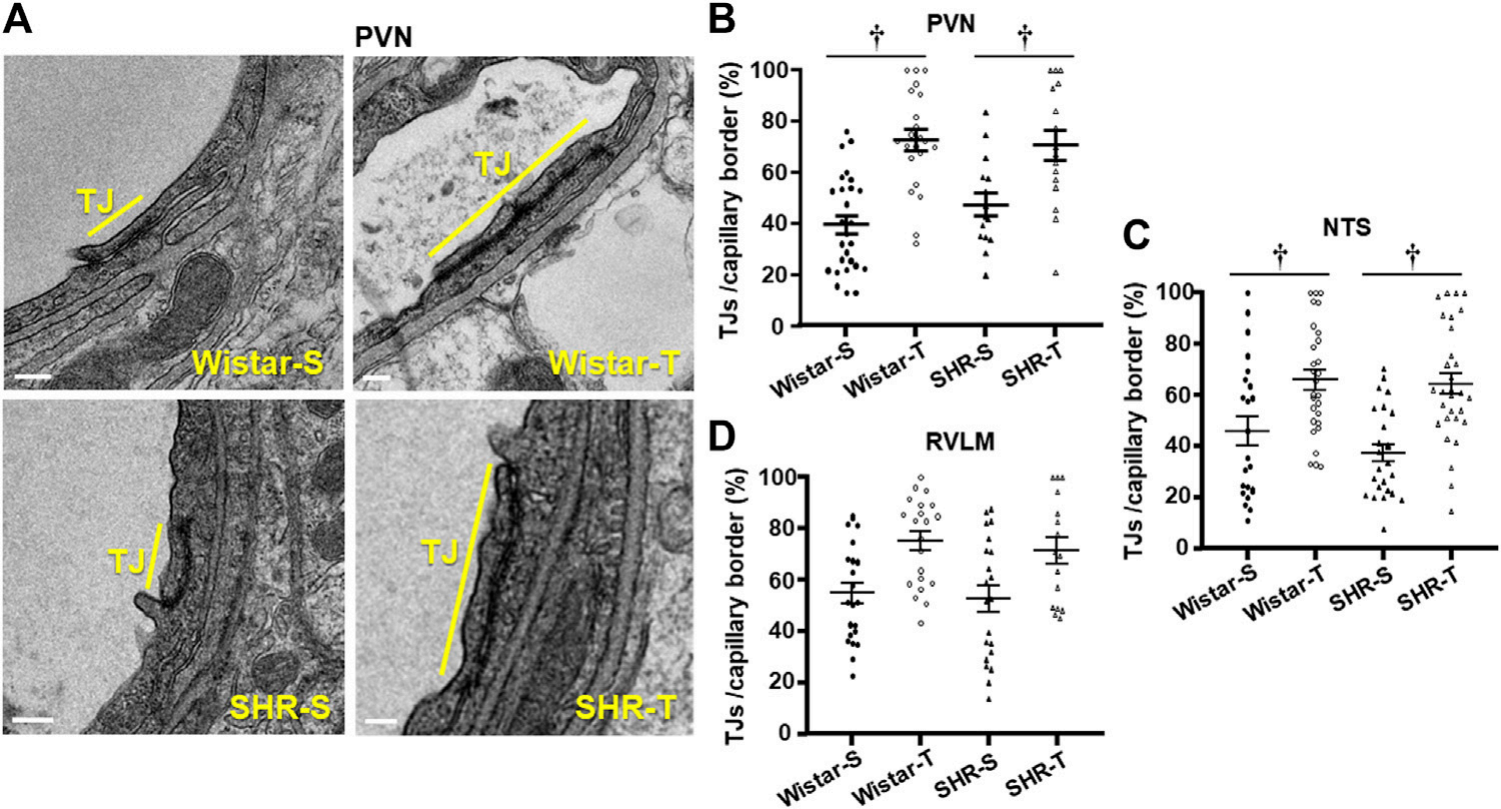
Effects of hypertension and exercise training on tight junction (TJ) occupancy of capillary border. **(A)**. Electron micrographs depicting TJ extension (yellow bars) in the border of neighboring endothelial cells within PVN capillaries of sedentary (S) and trained (T) SHR and Wistar rats. Scale bars = 200 nm. Right panels show the quantification of TJ extension in the 4 experimental groups at the end of protocols within the PVN **(B)**, NTS **(C)**, and RVLM **(D)** capillaries. *n* = 5–7 capillaries/rat, 3 rats/group. Comparisons made by 2-way factorial ANOVA. Significance (*p* < 0.05) is † vs. respective S control.

The occurrence, density and extension of TJs within capillary borders were also quantified as indexes of possible hypertension- and exercise-induced changes in the paracellular transport. Within the three areas analyzed, the number of TJ/capillary (on average 1.04 ± 0.09/capillary) was not changed by hypertension or exercise training. There were slight changes in capillary border extension and TJs extension within the three areas analyzed (values on Table 2). To analyze the effects of hypertension and exercise on TJ it is important to consider both, that is TJ extension to capillary border extension ratio. Figure 4A depicts representative photomicrographs of PVN capillaries of the four experimental groups comparing the percentage of occupancy of the capillary border by the TJ. Quantitative data (Figure 4B) confirmed that TJ extension/capillary border extension was not changed by hypertension within the 3 autonomic areas. In contrast, trained exhibited significant increases in TJ occupancy of the capillary border within the PVN (Wistar-T = +78%, SHR-T = +43%, Figures 4A, B) and NTS (Wistar-T = +44%. SHR-T = +72%, Figure 4C; Supplementary Figure S2), with a slight not significant increase in the RVLM (Wistar-T = +37%, SHR-T = +36%, Figure 4D; Supplementary Figure S2).

Consequently, TJ extension/capillary border was not changed by hypertension, but significantly increased in both trained groups Quantitative data (Figure 4B) confirmed that exercise training greatly increased TJ/capillary border by 78% and 43% (Wistar-T and SHR-T, vs. Wistar-S and Wistar-S, respectively), without significant change in SHR-S compared to Wistar-S. Similar hypertension- and exercise-induced effects were observed in the NTS and RVLM (Table 2; Figures 4C, D).

**FIGURE 5:**
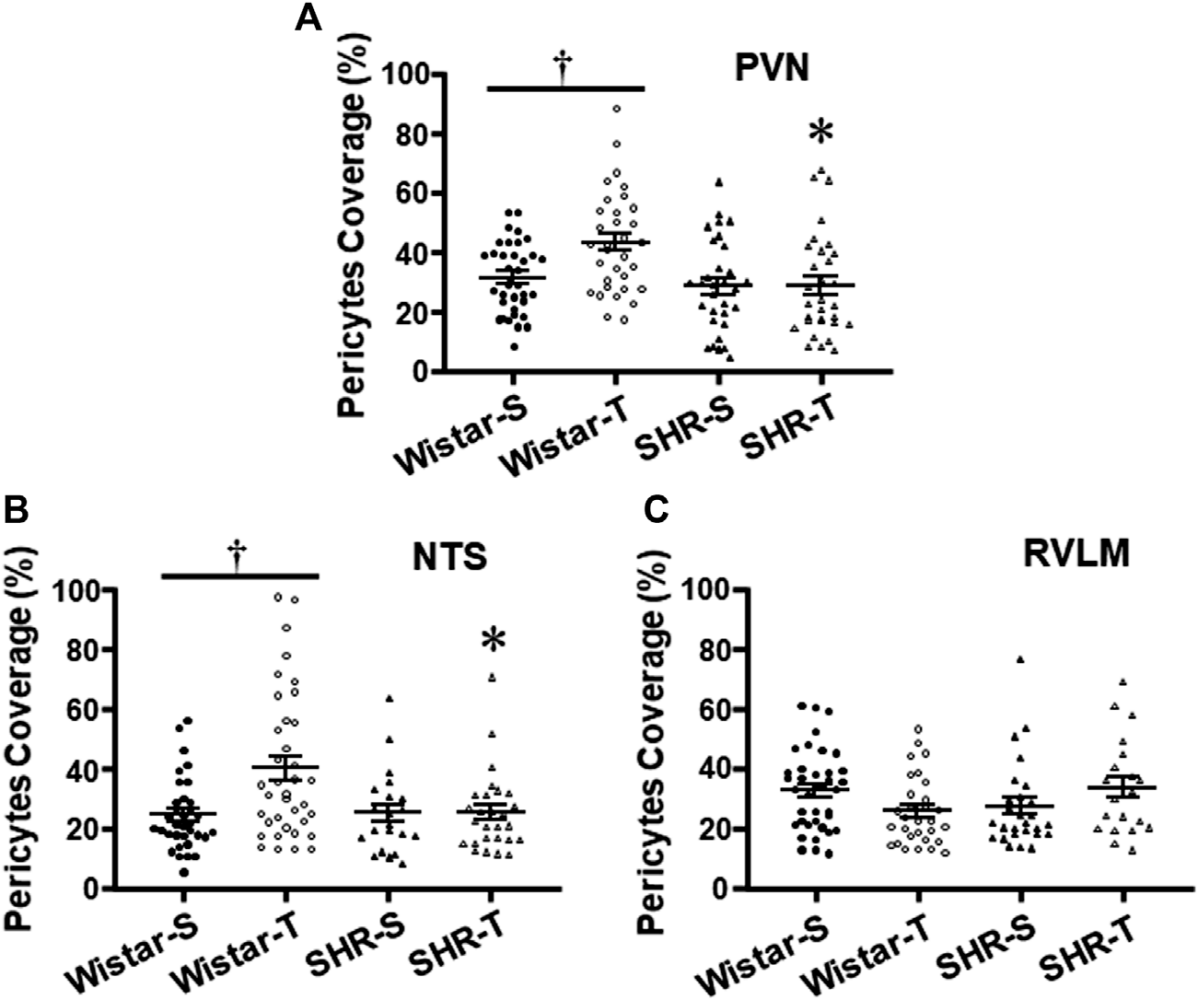
Effects of hypertension and exercise training on pericytes coverage of endothelial cells within the PVN **(A)**, NTS **(B)**,and RVLM **(C)** capillaries. *n* = 5–11 capillaries/area/rat, 3 rats/group. Comparisons made by two-way factorial ANOVA. Significances (*p* < 0.05) are * vs. respective Wistar group; † vs. respective S control.

Ultrastructural analyses of pericytes length in relation to capillary abluminal perimeter revealed that except for the RVLM (Figure 5C), exercise training increased pericytes’ capillary coverage within the PVN and NTS of normotensive rats, an effect that was abrogated by hypertension (Table 2; Figures 5B, C).

### 3.4 Hypertension- and training-induced changes in transcellular vesicle number correlate with BBB permeability while BBB permeability changes correlate with worsening and improvement, respectively, of the autonomic control of the circulation

**FIGURE 6:**
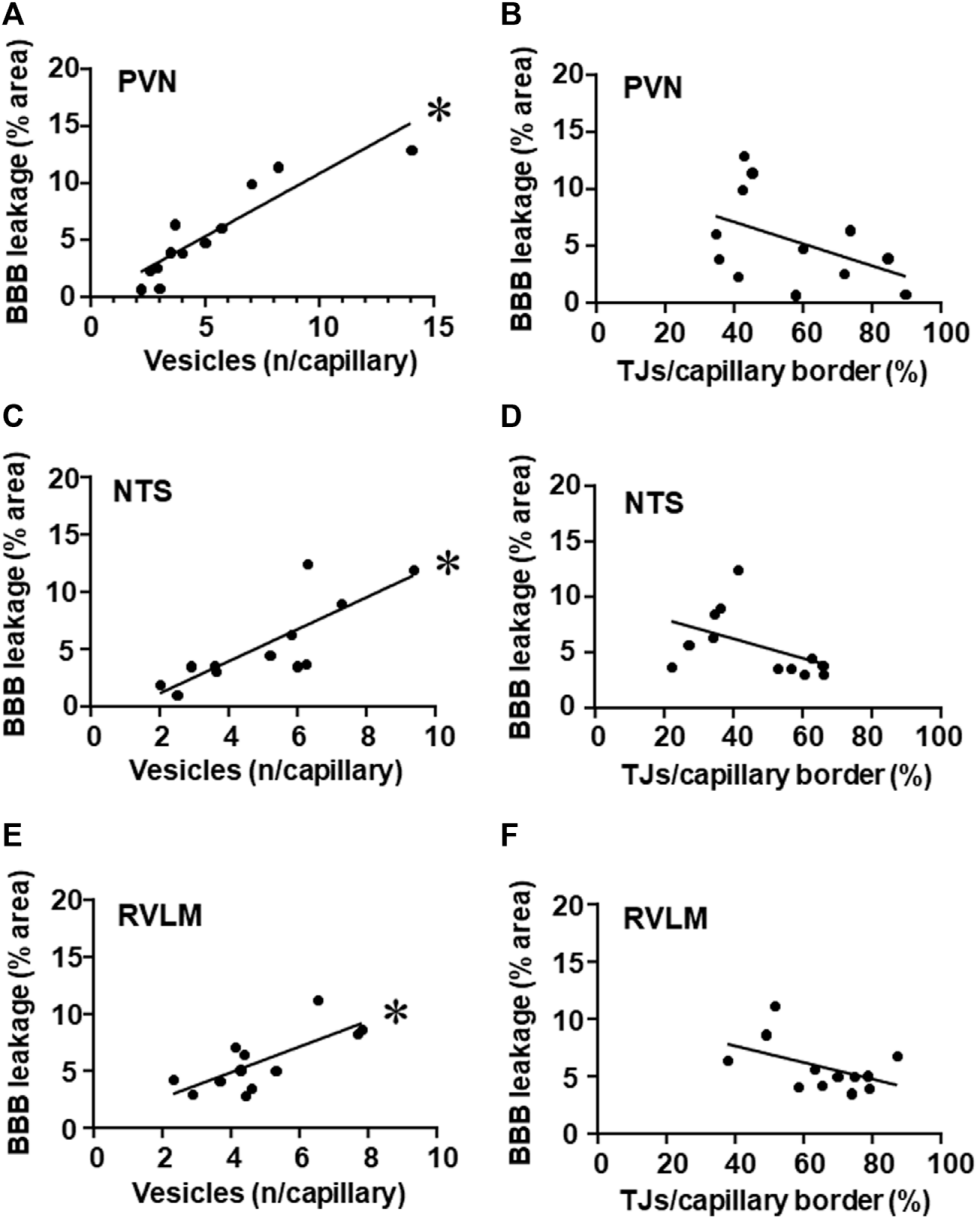
Correlations between the number of vesicles/capillary and BBB permeability **(A,C,E)** and between tight junctions/capillary and BBB permeability **(B,D,F)** within the PVN **(A,B)**, NTS **(C,D)** and RVLM **(E,F)** of sedentary and trained SHR and Wistar rats. BBB permeability and respective vesicles/capillary values, were obtained in 3 rats/group. Linear regression equations, correlation coefficients and *p* values are: PVN vesicles x BBB permeability Y = 1.10x–0.16, *r* = 0.905, *p* < 0.001; NTS vesicles x BBB permeability Y = 1.40x–1.66, *r* = 0.808, *p* = 0.002; RVLM vesicles x BBB permeability Y = 1.12x + 0.44, *r* = 0.743, *p* = 0.006; PVN TJs/capillary border x BBB permeability Y = −0.10x +10.9, *r* = − 0.461, *p* = 0.131; NTS TJs/capillary border x BBB permeability Y = −0.09x +9.7, *r* = −0.469, *p* = 0.124; RVLM TJs/capillary border x BBB permeability Y = −0.07x +10.7, *r* = −0.479, *p* = 0.229. * denotes a significant correlation.

It is important to note that, within the three autonomic areas, BBB permeability changes showed strong positive correlations with vesicle number/capillary. While hypertension-induced augmentation of vesicles counting was accompanied by increased BBB leakage, training-induced reduction was accompanied by decreased BBB permeability within the PVN, NTS, and RVLM (Figures 6A, C, E, respectively). Notice that, except for TJ extension, exercise training did not change BBB leakage, vesicle counting and autonomic function in normotensive groups. On the other hand, the mild negative correlations between tight junctions’ extension/capillary border (exercise dependent but hypertension-independent) and BBB leakage observed in the PVN, NTS, and RVLM did not attain significance (Figures 6B, D, F).

Since the increased vesicles ‘counting, the augmented BBB leakage and the autonomic dysfunction were both hypertension- and exercise-dependent, we also correlated the observed BBB permeability changes within the PVN, NTS, and RVLM with recorded changes in SAP and PI variabilities and their spectral components. In all autonomic areas, BBB leakage changes exhibited strong positive correlations with both SAP variability and sympathetic vasomotor activity changes (regression equations, *r* and *p* values on Table 3). On the other hand, BBB permeability was negatively correlated with both PI variability and parasympathetic modulation of the heart (Table 3).

**TABLE 3:** Linear regression equations, coefficients (r) and *p* values correlating BBB permeability with autonomic parameters in PVN, NTS, and RVLM capillaries of sedentary and trained SHR and normotensive controls.

	Regression equation	*r*	*p*-Value
BBB permeability x SAP variability			
PVN	Y = 2.55 x + 14.91*	0.761	0.006
NTS	Y = 3.17 x + 10.94*	0.776	0.004
RVLM	Y = 3.85 x + 5.50*	0.617	0.014
**BBB permeability x LF-SAP**			
PVN	Y = 0.47 x + 4.61*	0.697	0.003
NTS	Y = 0.67 x + 3.39*	0.822	<0.001
RVLM	Y = 0.65 x +3.13*	0.604	0.013
**BBB permeability x PI variability**			
PVN	Y = −1.82 x + 38.74*	−0.567	0.022
NTS	Y = −2.46 x + 42.63*	−0.628	0.009
RVLM	Y = −3.16 x + 48.03*	−0.607	0.013
**BBB permeability x HF-PI**			
PVN	Y = −0.53x +10.63*	−0.714	0.002
NTS	Y = −0.59 x +11.11*	−0.656	0.006
RVLM	Y = −0.90 x +13.20*	−0.744	<0.001

BBB, blood-brain barrier; HF, high frequency component; LF, low frequency component; PI, pulse interval; SAP, systolic arterial pressure; BBB, permeability and respective autonomic parameters values, 4 rats/group, were used to obtain the linear regression equations. * denotes a significant correlation.

## 4 Discussion

The present set of data corroborate previous observations on BBB leakage and autonomic dysfunction in chronically hypertensive rats and the correction of both by exercise training. Additionally, new original observations were made: 1) The increased formation of transcellular vesicles, not changes in paracellular transport, was accompanied by augmented BBB leakage within autonomic areas of the sedentary SHR; 2) Exercise training normalizes the increased SHR absorptive transcytosis by a marked decrease in transcellular vesicle formation in these areas; 3) Decreased vesicle number/capillary in trained SHR is positively correlated with exercise-induced reduction of BBB permeability within the PVN, NTS, and RVLM; coherently, correction of BBB leakage is strongly correlated with the improvement of autonomic control of the circulation observed in the trained SHR; 4) Hypertension does not change while exercise augments TJ density in both SHR and Wistar trained groups, potentially improving its tightness; 5) Exercise training also increases pericytes’ coverage of endothelial cells in normotensive rats, an effect abrogated by hypertension.

It is well known that intact BBB ensures an efficient selective barrier between the circulating blood and the central nervous system, guarantying a constant rate of neuronal signaling and homeostasis (Abbott et al., 2006; Daneman and Prat, 2015; Haseloff et al., 2015; Ayloo and Gu, 2019; Greene et al., 2019). The loss of BBB integrity in pathological conditions such as neurodegenerative diseases, stroke, trauma, e.g., Leads to dysfunction of both transcellular vesicle trafficking and fluid homeostasis facilitating the central nervous system entry of plasma and serum proteins, inflammatory mediators, circulating toxins, and macrophages that culminate in strong neuroinflammation, altered neuronal signaling and an inadequate homeostasis (Zlokovic, 2008). Most of these effects have been attributed to TJ breakdown. Low-grade inflammation, altered neuronal signaling and deficient homeostasis are also observed in chronic hypertension (Shi et al., 2010; Abboud et al., 2012; Masson et al., 2014). Conflicting observations are reported for dysfunctional BBB in hypertensive animals including TJs’ breakdown (Jiao et al., 2011; Pelisch et al., 2013; Mohammadi and Dehghani, 2014; Guo et al., 2019) as well as intact TJs with compromised transcellular transport within the cortex, hippocampus and striatum (Ueno et al., 2004; Krueger et al., 2013). Analyzing changes in caveolin-1 expression within the PVN of the SHR we previously suggested that loss of BBB integrity and its correction by exercise training were due to increased transcytosis and exercise-induced correction, respectively, without changes in the paracellular transport (Fragas et al., 2021). In the present study, by comparing leakage changes, vesicles/capillary, TJs/capillary border and functional parameters in sedentary and trained SHR, we uncover the primary mechanism regulating BBB leakage/correction within the PVN, NTS and RVLM of hypertensive rats: Changes in the endothelial vesicle formation, the so-called *absorptive transcytosis*, which is increased by hypertension and normalized after exercise training. There was also a tendency for reduced BBB leakage in the presence of exercise-induced increase in TJs extension/capillary border, but it did not attain significance suggesting that paracellular transport was not the driving mechanism. Hypertension-induced upregulation of the vasoconstrictor axis of the renin-angiotensin system within autonomic areas is accompanied by either large increase in local angiotensin II availability and important autonomic imbalance (Masson et al., 2014; Chaar et al., 2015; Raquel et al., 2022). Biancardi et al. (2014) also demonstrated in SHR that circulating angiotensin II leaks *via* BBB lesion gaining access to hypothalamic and brain stem neurons and microglial cells. The additional entrance of plasma angiotensin II, *via* absorptive transcytosis, augmented its availability within the PVN, NTS, and RVLM and potentiated the autonomic dysfunction, as recorded in SHR-S rats. Indeed, Pyner (2021) and Shanks and Ramchandra (2021) reviewing previous studies clearly demonstrated that within the autonomic areas the augmented angiotensin II content activates premotor sympathetic and impairs parasympathetic neurons. It is important to note that increased hypertension-induced absorptive transcytosis was reduced and completely normalized after exercise training (Buttler et al., 2017; Fragas et al., 2021), as was the brain renin-angiotensin system hyperactivity (Masson et al., 2014; Chaar et al., 2015; Raquel et al., 2022). These changes were accompanied by decreased sympathetic and increased parasympathetic modulation of heart and vessels in the trained SHR. In the last years our laboratory accumulated evidence showing that more than blood pressure drop, exercise training is highly efficient to reduce peripheral sympathetic activity and augment cardiac vagal activity, which increases heart rate variability and reduces the elevated pressure variability, the main cause of end-organ damage in hypertension (Masson et al., 2014; Chaar et al., 2015; Buttler et al., 2017; Rocha-Santos et al., 2020; Fragas et al., 2021). Indeed, exercise-induced pressure reduction in hypertensive rats accounts for only 9%, while autonomic parameters changes are 3–9 times higher (values in Table 1). The present set of data proving the strict correlations between vesicle number/capillary, BBB permeability and autonomic control of the circulation emphasizes that exercise-induced normalization of barrier permeability by reducing angiotensin II availability also contributes to the beneficial effects of exercise training on autonomic modulation in the trained SHR. To our knowledge, our findings are the first to demonstrate that augmented absorptive transcytosis in hypertensive rats is restored by exercise training even in the persistence of hypertension. In a recent review Ayloo and Gu (2019) reported that transcytosis at the BBB is an active process that is dynamically regulated during development as well as during the establishment of diseases. Our results confirmed Ayloo and Gu’s observations in hypertension and indicated that transcytosis is also dynamically regulated by exercise training.

Other original observations of the present study were that hypertension did not change TJs’ expression, suggestive of unchanged paracellular transport, and that exercise training strengthened TJs’ density by augmented their occupancy of the interface of neighboring endothelial cells. TJs, constituted by trans interactions of transmembrane proteins (claudin-5, occludin and junctional adhesion molecules) linked to the cytoskeleton by zonula occludens complexes, are the major physical barrier for maintaining the structural integrity of vasculature and limiting paracellular diffusion of small solutes, ions and water across the BBB (Daneman and Prat, 2015; Greene et al., 2019). Interestingly, hypertension did not change TJs’ structure while exercise training increased its tightness. In a previous paper we reported that exercise training augmented claudin-5 expression, which plays an important role in the paracellular barrier to small molecules, suggesting an improvement in BBB selectivity (Fragas et al., 2021). Indeed, claudin-5 knockout in mice allowed rapid extravasation of tracers with molecular weights <800 Da from the intravascular compartment to the brain parenchyma (Nitta et al., 2003). Souza et al. (2017) also demonstrated in autoimmune encephalomyelitis that physical exercise restored the expression of claudins and occludin in the spinal cord and inhibited the local production of either reactive oxygen species and pro-inflammatory cytokines. The specific mechanism by which exercise training increased TJs tightness and its functional consequence on BBB selectivity remains to be determined in the trained SHR.

Indeed, factor(s) underlying the exercise-induced normalization of the increased BBB permeability in trained SHR is (are) not known. One possibility is the involvement of the major facilitator superfamily domain containing protein-2a (Mfsd2a) in transcytosis suppression (Ben-Zvi et al., 2014; Nguyen et al., 2014). Mfsd2a, a transmembrane lipid-transporter of docosahexaenoic acid largely expressed only in brain capillaries exhibiting BBB properties (not in circumventricular organs’ capillaries, neurons, pericytes and astrocytes (Ben-Zvi et al., 2014; Nguyen et al., 2014), has been shown to displace caveolin-1 from the plasma membrane thereby decreasing the formation of transcytotic vesicles (Li et al., 2007). Mfsd2a knockout mice exhibited robust leakage of plasma constituents into the brain parenchyma (Ben-Zvi et al., 2014; Nguyen et al., 2014). Moreover, Andreone et al. (2017) and Ocak et al. (2018) showed that Mfsd2a specifically inhibits caveolae-mediated transcytosis, playing a critical role in the maintenance of BBB integrity. Ongoing experiments in our laboratory are now investigating the role played by Mfsd2a in the exercise-induced correction of increased absorptive transcytosis that characterizes the chronic hypertension.

Another original observation of the present study was that exercise training increased pericytes’ coverage of the endothelial cell within the PVN and NTS capillaries of normotensive rats, an effect blocked by hypertension. Pericytes and endothelial cells, surrounded by the basement membrane, exhibit extensive signaling between them (Armulik et al., 2010). Indeed, an increased BBB leakage was shown in pericytes-deficient mice (Armulik et al., 2010; Bell et al., 2010). Future studies are needed to identify the mechanism(s) determining exercise-induced effect in normotensive rats and why this pericytes’ response is abrogated by hypertension.

## 5 Conclusion

The present set of data extends our knowledge on important mechanisms regulating BBB transport of substances between the circulating blood and the central nervous system. By evaluating changes in transcellular vesicle trafficking and showing its strong correlations with BBB leakage and autonomic control of the circulation, data indicate that absorptive transcytosis, not the paracellular transport, is the primary mechanism underlying both hypertension-induced dysfunction and exercise-induced correction of the BBB leakage. The normalized BBB permeability in the trained SHR contributes to facilitate the increase in heart rate variability and the decrease in pressure variability, important factors to reduce the occurrence of end-organ damage in the presence of hypertension. Data also reveal that TJ density is not altered by hypertension but TJ extension/capillary is largely increased by exercise training in both strains, a mechanism that strengths the resistance and controls the selectivity of the paracellular pathway. Together our results indicate that exercise training is an efficient therapy to correct the elevated BBB permeability, thus contributing to augment baroreceptor reflex sensitivity and to improve the autonomic control of the circulation even in the presence of hypertension.

## Data Availability

The original contributions presented in the study are included in the article/Supplementary Material, further inquiries can be directed to the corresponding author.
