# Supplementary figures and images for: Trained hypertensive rats exhibit decreased transcellular vesicle trafficking, increased tight junctions’ density, restored blood-brain barrier permeability and normalized autonomic control of the circulation
Source: Front Physiol. 2023 Feb 23;14:1069485. doi: 10.3389/fphys.2023.1069485 (PMC9997677; doi:10.3389/fphys.2023.1069485)

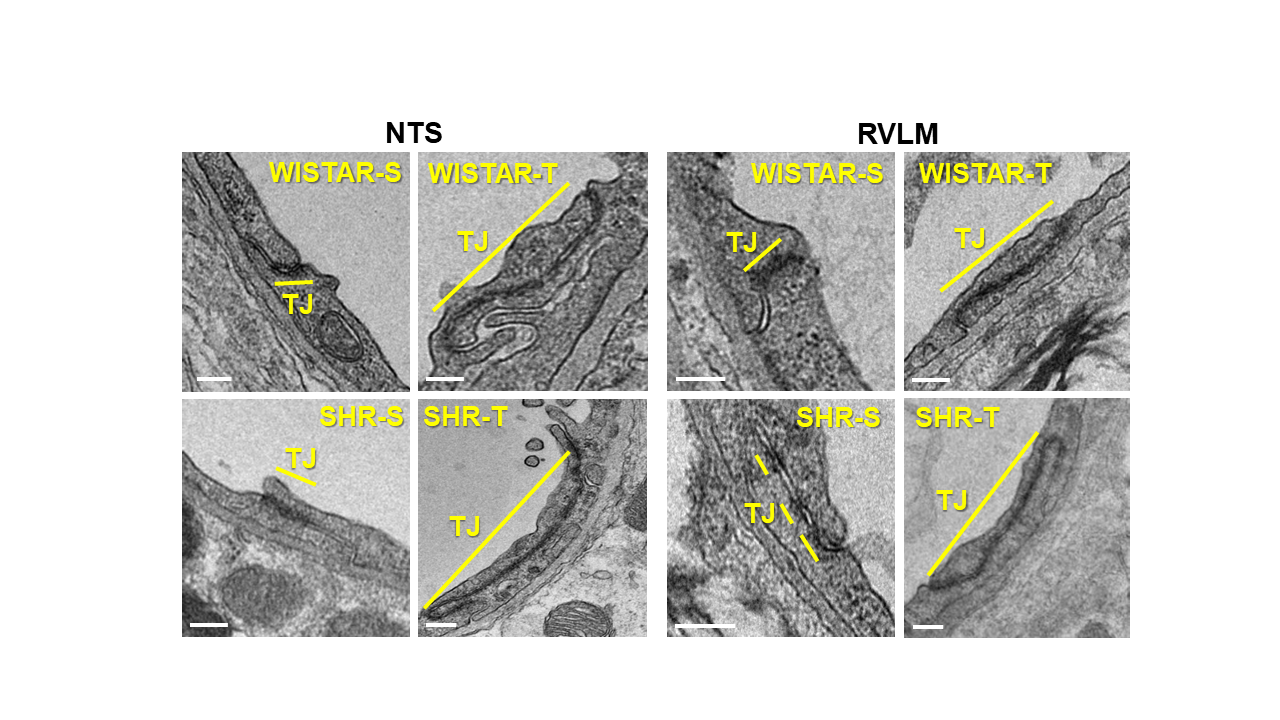

Supplement: Supplementary file 1 [file Image2.TIF]

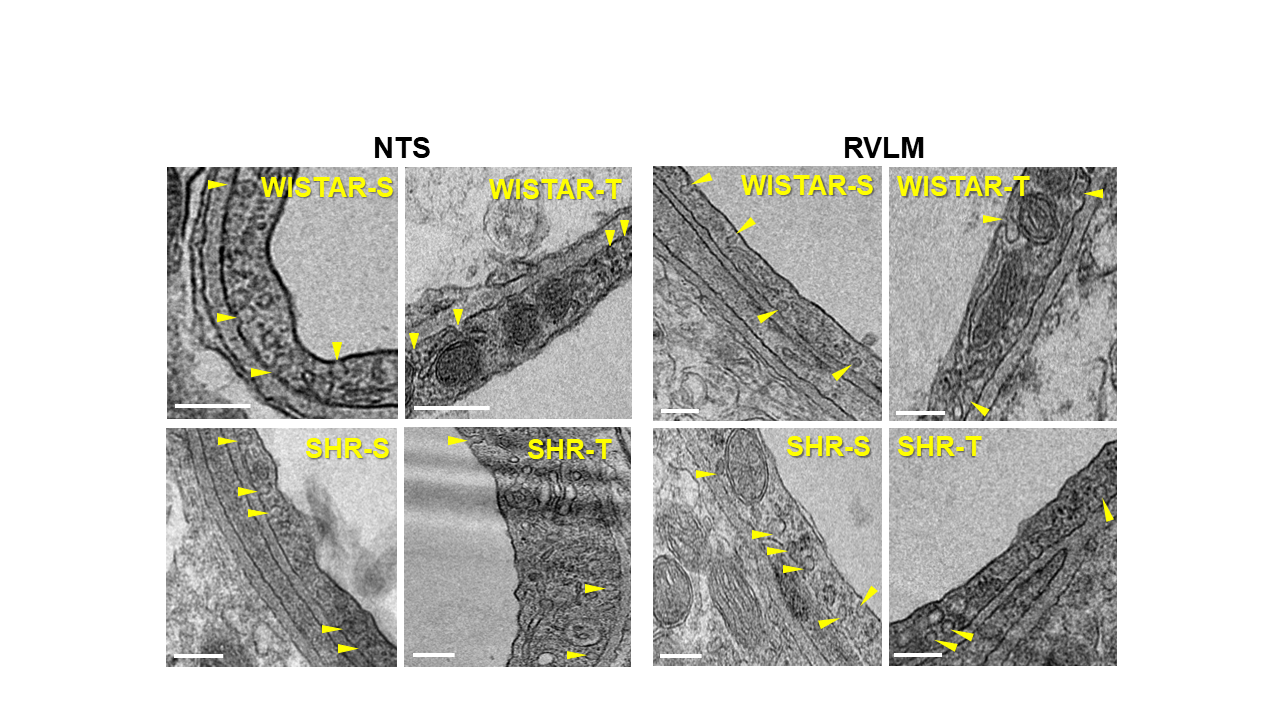

Supplement: Supplementary file 2 [file Image1.TIF]
