# Supplementary material for: Trained hypertensive rats exhibit decreased transcellular vesicle trafficking, increased tight junctions’ density, restored blood-brain barrier permeability and normalized autonomic control of the circulation
Source: Front Physiol. 2023 Feb 23;14:1069485. doi: 10.3389/fphys.2023.1069485 (PMC9997677; doi:10.3389/fphys.2023.1069485)

Data Supplement

Trained hypertensive rats exhibit decreased transcellular vesicle trafficking, increased tight junctions' density, restored blood-brain barrier permeability and normalized autonomic control of the circulation

Vanessa Brito Candido[1], Sany Martins Perego[1], Alexandre Ceroni[1], Martin A. Metzger[1], Alison Colquhoun[2], Lisete C. Michelini[1*]

From [1] Department of Physiology and Biophysics and [2] Department of Cell and Developmental Biology, Institute of Biomedical Sciences, University of Sao Paulo, São Paulo, SP Brazil

Running Head: ***Transcytosis changes in hypertension and exercise***

Corresponding Author: [*]Lisete C. Michelini, Ph.D,
ORCID: 0000-0003-2978-5406
Department of Physiology & Biophysics
Institute of Biomedical Sciences
University of Sao Paulo
Av. Prof. Lineu Prestes, 1524
05508-000 Sao Paulo, SP, Brazil
Phone: 55-11-3091.7213
e-mail: michelin@usp.br

SUPPLEMENTAL FIGURES CAPTIONS

Figure S1. Effects of hypertension and exercise training on transcellular vesicles number/capillary. Electron micrographs depicting the transcellular vesicles (yellow arrows) being formed in the luminal and abluminal borders of the endothelial cell within the NTS and RVLM capillaries of sedentary (S) and trained (T) SHR and Wistar rats. *n*=9-11 capillaries/rat, 3 rats/group. Scale bars = 200 nm

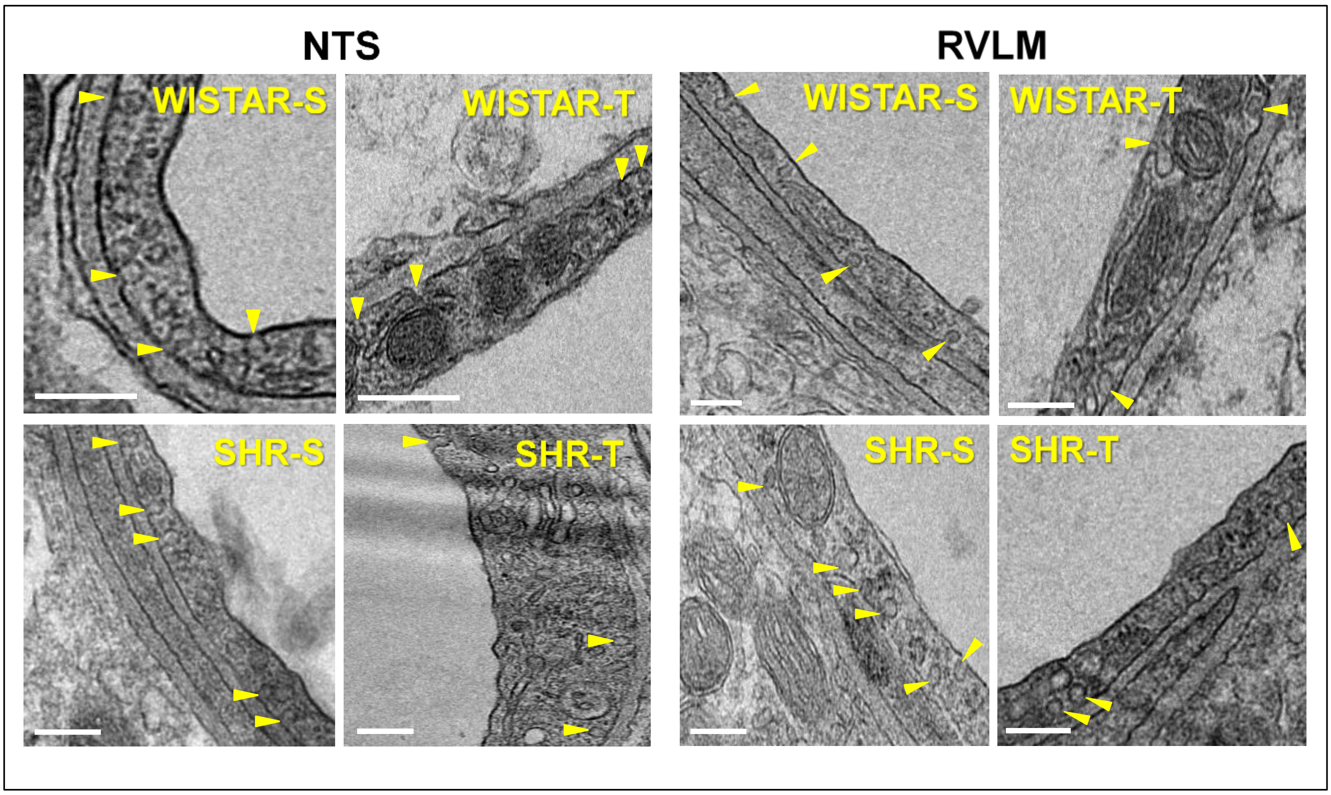

Figure S2. Effects of hypertension and exercise training on tight junction (TJ) occupancy of capillary border. Electron micrographs depicting TJ extension (yellow bars) in the border of neighboring endothelial cells within the NTS and RVLM capillaries of sedentary (S) and trained (T) SHR and Wistar rats. *n*=5-7 capillaries/rat, 3 rats/group. Scale bars = 200 nm.

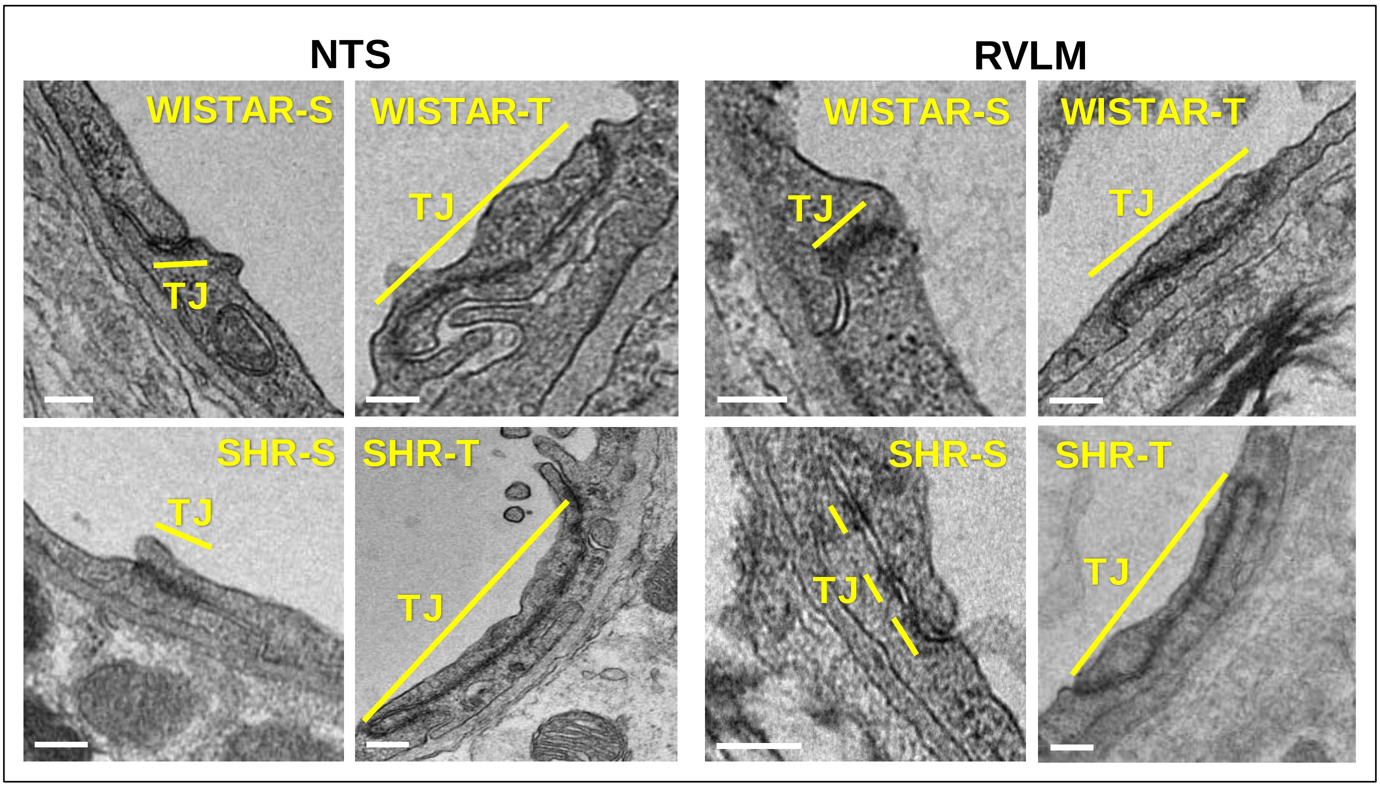

Supplement: Supplementary file 3 [file DataSheet1.PDF]
